# Evaluation of antifungal activity of glycoalkaloids from the *Solanum lycocarpum *St. Hil (lobeira) in the cell membrane of dermatophyte of *Trichophyton rubrum*

**DOI:** 10.1186/1753-6561-8-S4-P11

**Published:** 2014-10-01

**Authors:** Bruna AM Cantelli, Flavia R Barbosa, Tamires A Bitencourt, Mariza A Miranda, Jairo K Bastos, Mozart Marins, Ana Lucia Fachin, Mariana H De Abreu, Yasmin D Crivelenti, Thaís B Mesquita

**Affiliations:** 1 Unidade de Biotecnologia, Universidade de Ribeirão Preto (UNAERP), Av. Costábile Romano, 2.201 Ribeirania, Ribeirão Preto, CEP14096-900, Brazil; 2 Laboratório de Farmacognosia, Faculdade de Ciências Farmacêuticas de Ribeirão Preto, University of São Paulo (USP-FCFRP), Av. do Café, s/nº, Campus Universitário, Ribeirão Preto, São Paulo,14040-903, Brazil

## Background

The dermatophytes belong to one of the main groups of pathogenic fungus, characterized by the use of the host's keratin for its nutrition, which are the most common cause of fungal infection in the world, affecting millions of individuals annually, causing a huge economic impact [1]. Therefore, there was an increase in the search for new antifungal agents from natural sources, because the majority of the available drugs in the market presents a restricted number of cellular targets and there are reports of resistant fungal strain to these utilized drugs [2]. Glycoalkaloids from the *Solanum lycocarpum *plant (lobeira) presents several biological activities, such as cytotoxic and antimicrobial activities [3]. The goal of this work was determination the Minimum Inhibitory Concentration (MIC) of the solanine, solamargine and solasodine from the *S. lycocarpum *in addition to evaluate the effect of these alkaloids in the regeneration of the *Trichophyton rubrum *cell wall.

## Methods

The minimum inhibitory concentration (MIC) of alkaloids toward the ATTC MYA-3108 *T.rubrum *strain was determined by microdilution assay in 96-well plates using RPMI medium according to the protocol NCCLS M-38 (2002) for 7 days at 37C [4], using amphotericin B as control. The protoplast regeneration assay has been used to evaluate the level of plasma membrane damage caused by antifungal compounds [5]. Protoplasts were obtained from of MYA-3108 *T. rubrum *strain grown for 7 days. Regenerated protoplasts were selected on solid minimal medium, supplemented with 1 M sucrose, 0.2% casein and 0.07 mM NaNO_3 _by incubation with the MIC and 0.5 MIC concentrations of alkaloids for 7 days at 28°C.

## Results and conclusion

The results indicated that the glycoalkaloids solamargine, solasodine and solanine presented a MIC of 3.12 µg/mL, 12.5 µg/mL and >25 µg/mL, respectively, the amphotericin B control presented a MIC of 0.39 µg/mL. The same concentrations of the three glycoalkaloids were tested and didn't inhibit the protoplasts regeneration and also didn't cause reduction on the size of the colonies compared with the aculeacin control that inhibited 100% of the regeneration. These data shows that the alkaloids solamargine and solanine presented pronunciated antifungal activity, but didn't act in the membrane of *T.rubrum*.
